# Room-Temperature Self-Healable and Mechanically Robust Thermoset Polymers for Healing Delamination and Recycling Carbon Fibers

**DOI:** 10.1021/acsami.1c16105

**Published:** 2021-10-27

**Authors:** Xiaming Feng, Guoqiang Li

**Affiliations:** Department of Mechanical & Industrial Engineering, Louisiana State University, Baton Rouge, Louisiana 70803, United States

**Keywords:** thermoset polymer, room-temperature self-healing, multiple hydrogen bonds, composite laminates, recyclability

## Abstract

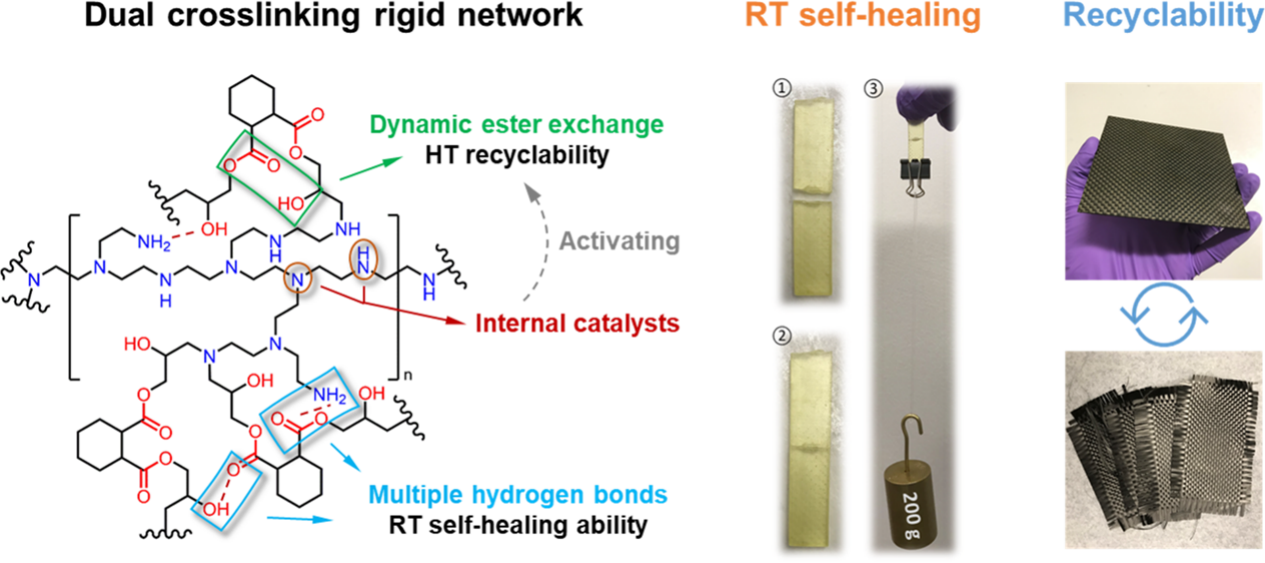

The advocacy of carbon neutrality and circular economy encourages people to pursue self-healing and recycling of glassy thermoset polymers in a more realistic and energy-saving manner, the best being intrinsic healing under room temperature. However, the high mechanical robustness and healing ability are mutually exclusive because of their completely opposite requirements for the mobility of the polymer networks. Here, we report a dual-cross-linked network by slightly coupling the low-molecular-weight branched polyethylenimine with an ester-containing epoxy monomer in a nonstoichiometric proportion. The highly mobile and dense noncovalent hydrogen bonds at the chain branches and ends can not only complement the mechanical robustness (tensile strength of 61.6 MPa, elastic modulus of 1.6 GPa, and toughness of 19.2 MJ/m^3^) but also endow the glassy thermoset polymer (*T*_g_ > 40 °C) with intrinsic self-healing ability (healing efficiency > 84%) at 20 °C. Moreover, the resultant covalent adaptive network makes the thermoset polymer stable to high temperatures and solvents, yet it is readily dissolved in ethylene glycol through internal catalyzed transesterification. The application to room temperature delamination healing and carbon fiber recycling was demonstrated as a proof-of-concept.

## 1 Introduction

The concept of carbon neutrality and circular economy is sweeping the world on account of the growing problems of oil crises and environmental issues. Self-healable and recyclable thermoset polymers are attracting tons of attention for their reliability and longevity in service.^1−4^ Due to the robust mechanical properties, excellent chemical resistances, and thermal stability, glassy thermoset polymers have been applied as structural materials in a wide range of load-carrying structures.^5−7^ The earlier generation of self-healing glassy thermoset polymer materials relies on the incorporation of external healing agents,^8^ such as microcapsules^9^ and microvascular networks^10^ containing reactive monomers. The healing process can be varied from cold to high temperatures depending on the reactivity of the healing agents. However, these kinds of self-healing materials have some inherent problems such as limited healing cycles, short shield life, high viscous precursor mixture, and degraded mechanical performance of resultant materials due to the addition of a large quantity of weak microcapsules, which make them difficult to fabricate high-performance self-healable structural composites with complex construction, such as laminates. Most importantly, they are not reprocessable due to the permanent cross-linked networks.

To overcome the abovementioned problems, the latest generation of self-healing glassy thermoset polymers introduce various dynamic covalent bonds within the cross-linked network.^11−17^ The resultant covalent adaptive network can reorganize by thermally or chemically triggering the reversible reactions and thus endowing the polymer with intrinsic multiple self-healing ability and recyclability. The rearrangement and reorganization of covalent adaptive networks heavily depend on both high molecular mobility and high activity of reversible bonds.^18,19^ The former makes cleaved moieties rapidly find their counterparts, and the latter can accelerate the formation of new bonds. Therefore, the self-healing process of all the reported cases requires thermal stimulus or solvent assistance to soften the rigid fractured networks first and then to heal. This is also why intrinsic room-temperature self-healing is usually observed in soft gels and elastomers but not in glassy thermoset polymers. Therefore, to easily implement self-healing during most practical applications, how to achieve intrinsic self-healing under room temperature for glassy thermoset polymers is a grand challenge.

So far, to our knowledge, only three papers have reported the intrinsic room-temperature self-healing of glassy polymers.^20−22^ They were prepared by noncovalently cross-linking the low-molecular-weight chains, such as linear poly(ether-thiourea)^20^ and polyurethane^22^ with the weak but dense hydrogen bonds, which can overcome the persistent barrier between high stiffness and self-healing ability. The resultant polymers are rigid yet repairable at room temperature. All these polymers still belong to the category of thermoplastics, which are unstable to thermal and chemical solvents. Moreover, the noncovalent cross-linking of networks always means low mechanical strength, and indeed, their tensile strengths are all below 40 MPa (tested at a stretching rate of 10 mm/min). These results demonstrate the limited application of these polymers in heavy load-carrying structures and harsh conditions.

**Figure 1:**
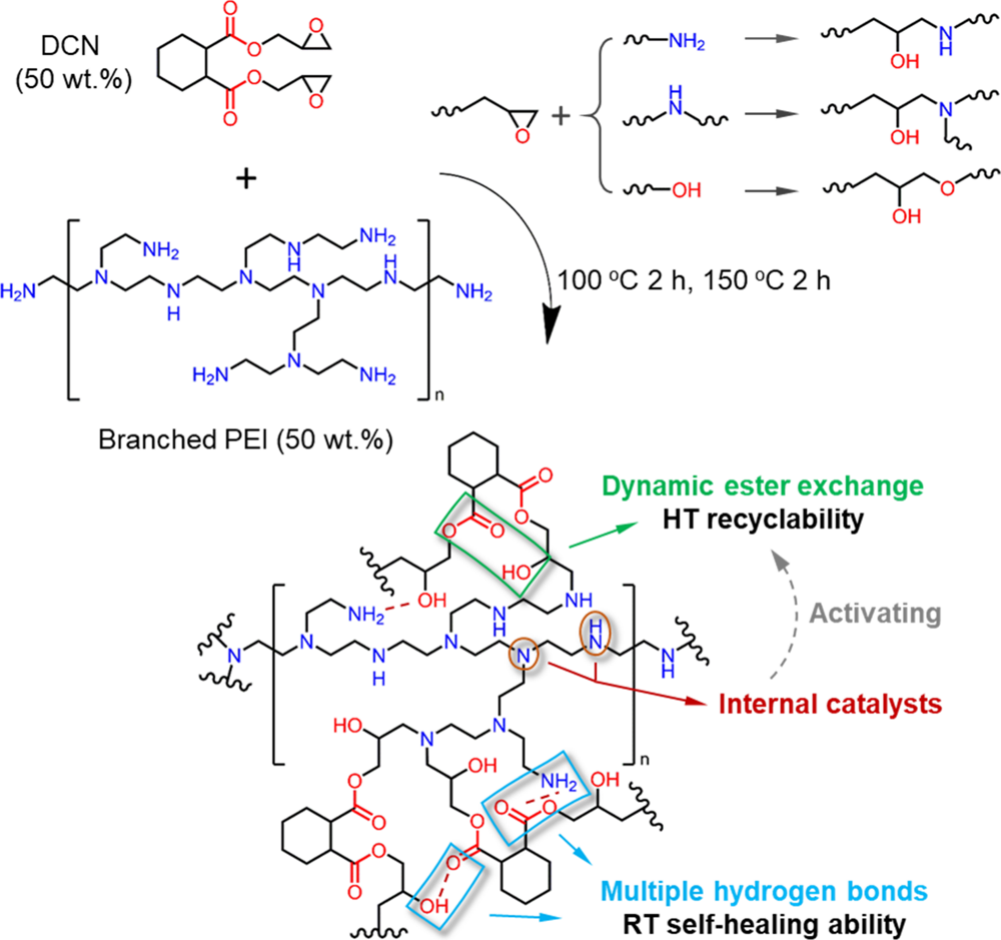
Synthesis route of the room-temperature self-healable thermoset DCN–PEI polymer in a mass ratio of 1/1.

Inspired by these works, here, we report a thermoset network that is covalently and mildly cross-linked from a low-molecular-weight branched polyethylenimine (PEI) and an ester-containing epoxy monomer (diglycidyl 1,2-cyclohexanedicarboxylate, DCN) in a nonstoichiometric proportion (Figure 1). Different from the well-established PEI cross-linked bisphenol A diglycidyl ether (DGEBA) epoxy monomer in a stoichiometric ratio,^23^ in which most of the amines/imines have been exhausted, the excess primary amine terminals and secondary amines on branched chains, the generated hydroxyls, and the inherent ester groups of the DCN–PEI network can form high-density but loosely packed multiple hydrogen bonds, which leads to complementary noncovalent cross-linking of the network. Due to the dual cross-linking actions, the resultant polymer is not only mechanically robust (tensile strength of 73.9 MPa and elastic modulus of 1.6 GPa, tested at a stretching rate of 10 mm/min) but also chemically and thermally stable. The high mobility of the chain branches and ends can accelerate the reconfiguration of the cleaved hydrogen bonds even at room temperature, namely room-temperature self-healing of the resultant glassy thermoset polymer. The recovery of the dense hydrogen bonds is enough to obtain acceptable healing efficiency (84.21%) even though a few broken covalent bonds are not restored. Moreover, the internal catalytic action of the tertiary amines within the network makes the thermoset polymer readily dissolved in alcohols through catalyst-free transesterification. As proof-of-concept studies, both glass fiber and carbon fiber reinforced laminated composites were prepared. The delamination healing of the glass fiber reinforced laminate at room temperature, and recycling of the carbon fibers in the carbon fiber reinforced laminate with mild recycling conditions were demonstrated.

## 2 Results and Discussion

### 2.1 Material Design and Characterization

The thermoset DCN–PEI polymer can be simply prepared by one-pot polycondensation of commercially available DCN and branched PEI monomers in a nonstoichiometric ratio (a mass ratio of 1/1) (Figure 1). First, the curing kinetics was studied by differential scanning calorimetry (DSC) at different heating rates (Figure S1). The broad exothermic peaks corresponding to the heat of solidification appeared from 60 to 100 °C, suggesting mild curing temperature and energy saving in real-world applications. In addition, the apparent activation energy (*E*_a_) was determined from the peak temperatures (*T*_p_) at different heating rates according to Kissinger’s method^24^ (Figure S2). The *E*_a_ value was calculated to be 63.5 kJ/mol, which is comparable to those of commonly used amine curing agents.^25^ These readily available monomers coupling with an easily achievable curing process make our DCN–PEI polymer have great potential for massive industrial production.

**Figure 2:**
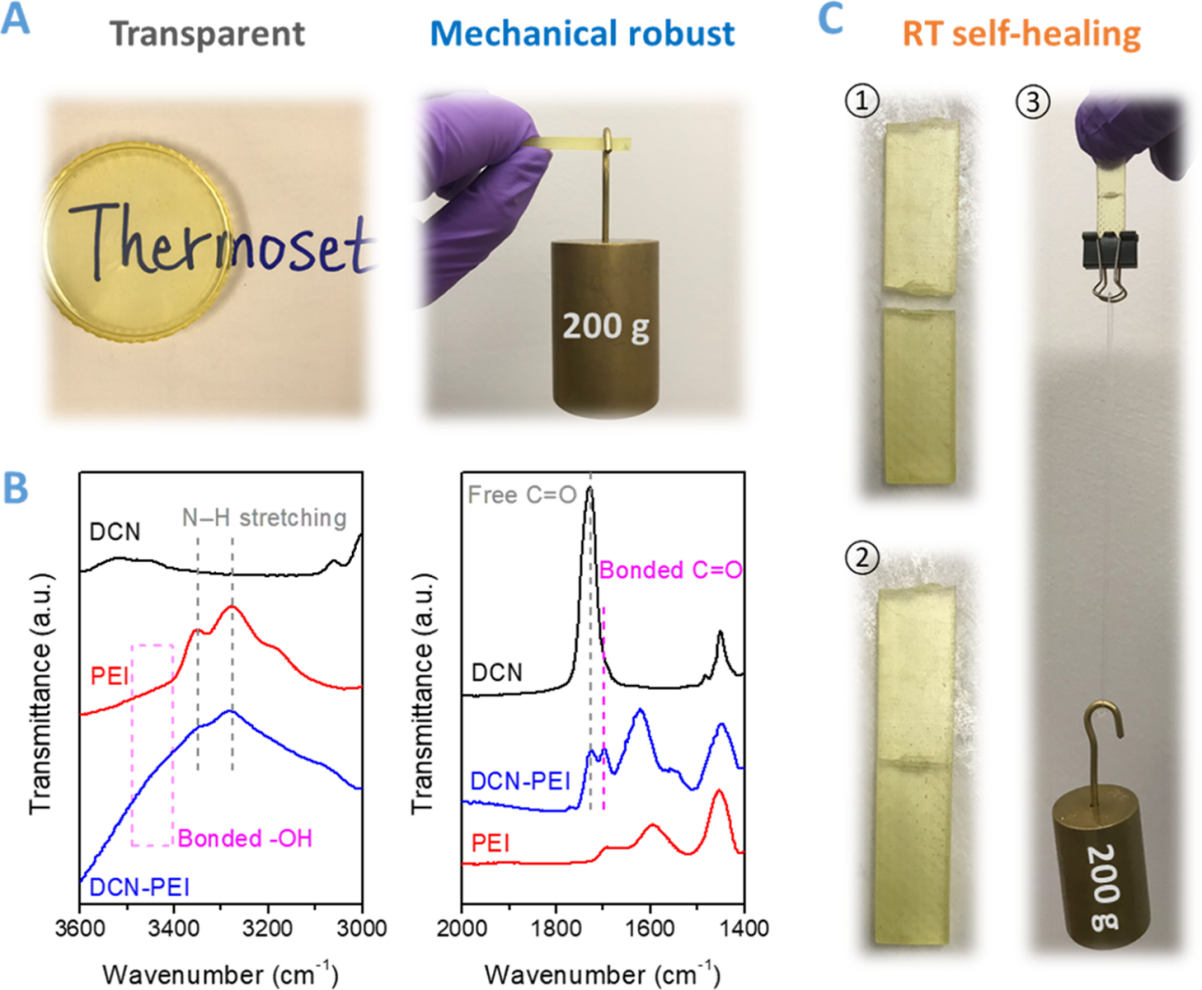
(A) Illustrations of transparency and mechanical toughness of the thermoset DCN–PEI polymer (RT: room temperature; HT: high temperature). (B) FTIR spectra of DCN, PEI, and the cross-linked DCN–PEI showing the hydrogen bonding interactions of −OH and C=O structures in the thermoset DCN–PEI network. (C) Photographs illustrating the room-temperature self-healing of DCN–PEI: (1) two separate pieces after fracturing, (2) the healed integral after 5 min at 20 °C, and (3) the state bearing a weight of 200 g.

FTIR spectra were conducted to assess the conversion degree of the epoxy group (Figure S3). The epoxy group centered at 902 cm^–1^ completely disappeared after thermal cross-linking, indicating the full curing of the epoxy group. As shown in Figure 2A, the resultant DCN–PEI polymer displays good transparency and mechanical robustness with a tensile Young’s modulus of 1.6 GPa, which can easily sustain a heavy load of 200 g without any bending in single cantilever mode at room temperature. The enlarged FTIR spectra clearly demonstrate the abundant hydrogen bonds ascribed to −NH, −OH, and C=O moieties in the thermoset DCN–PEI network^26,27^ (Figure 2B). Because the unique branched structure of PEI enables loose packing of adjacent main chains, the excess −NH groups on the movable side chains can assemble multiple hydrogen bonds within the network. These dense hydrogen bonding interactions and rigid hexatomic rings of DCN contribute to the excellent mechanical rigidity of the DCN–PEI polymer. Moreover, our thermoset DCN–PEI polymer exhibits a fantastic room-temperature self-healing property despite its rigid and covalently cross-linked nature. After manual compression of the low temperature (about −20 °C) fractured surfaces for 5 min at room temperature, the two broken pieces of a rectangular specimen can easily rejoin to lift a load of 200 g (Figure 2C).

**Figure 3:**
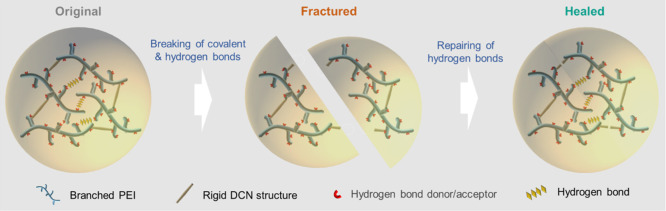
Schematic illustration of the fracture and self-healing behaviors of the DCN–PEI network showing the repairing of abundant hydrogen bonds from the movable moieties of the branched PEI at room temperature.

This room-temperature self-healing ability is attributed to the high mobility of assembled multiple hydrogen bonds associated with the side chains of PEI, which can make the damaged networks reconnect by the reconfiguration of dynamic hydrogen bonds even at temperatures below the glass transition temperature (*T*_g_) (Figure 3). It is worth mentioning that the DCN–PEI polymer is still tough even at −20 °C. It is difficult to create a smooth fractured surface that can perfectly align together, which makes the quantitative examination of the room-temperature self-healing ability of pure DCN–PEI difficult to achieve. This will be specifically discussed in the following section. Furthermore, the presence of β-hydroxyester and the internal catalytic action of the secondary/tertiary amines enable the thermoset DCN–PEI polymer to be readily dissolved in alcohols through the transesterification process at a mild temperature, which endows the DCN–PEI polymer with great potential in fabricating recyclable fiber reinforced composite laminates.

**Figure 4:**
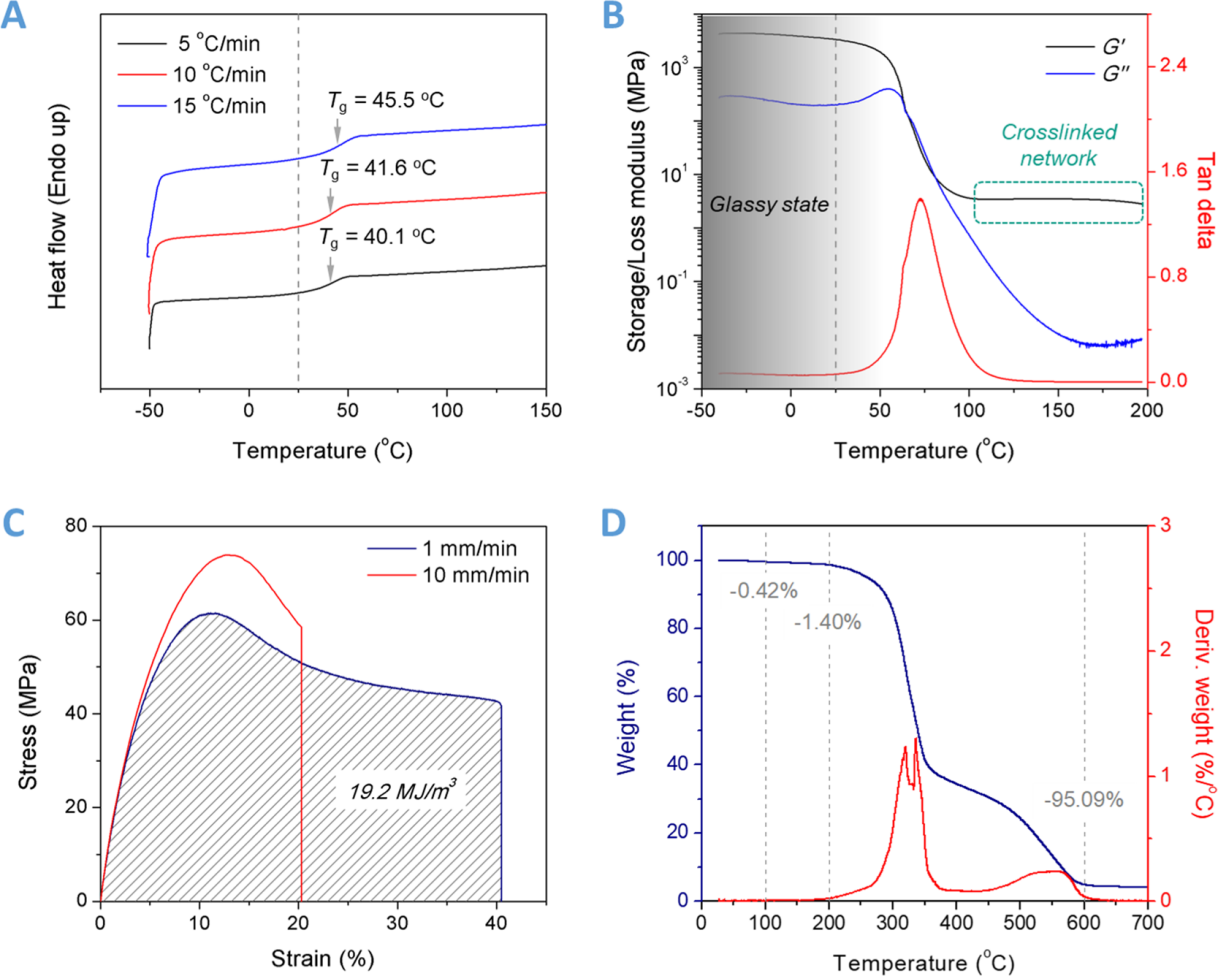
Characterization of the thermoset DCN–PEI polymer (a mass ratio of 1/1). (A) DSC profiles of the DCN–PEI polymer during second heating from −50 to 150 °C at different heating rates of 5, 10, and 15 °C/min. (B) Temperature dependence of the storage modulus, loss modulus, and loss angle of the DCN–PEI polymer at a heating rate of 3 °C/min and a frequency of 1 Hz. (C) Room-temperature stress–strain curves of the DCN–PEI polymer at stretching rates of 1 mm/min and 10 mm/min. (D) TG and derivative thermogravimetry (DTG) profiles of the DCN–PEI polymer at a heating rate of 10 °C/min under air atmosphere.

The *T*_g_ value of DCN–PEI was determined through DSC tests from −50 to 170 °C at different scan rates from 5 to 15 °C/min (Figure 4A). It increases as the heating process speeds up and varies from 40 to 45.5 °C, which indicates that DCN–PEI is a glassy polymer at ambient temperature. In addition, no melting-related peak appears in the tested temperature range, demonstrating the amorphous nature of the DCN–PEI polymer, which is in accordance with the high transparency. Moreover, as shown in Figure S4, the feed ratios of DCN and PEI monomers play an important role in *T*_g_ of resultant DCN–PEI polymers, which can vary from below room temperature to high temperature. Here, we fixed the ratio of DCN to PEI as 1/1. The optimized network reaches the balance between covalent cross-linking and noncovalent cross-linking by hydrogen bonding. It is rigid at room temperature, mechanically strong, and also has satisfied side chain mobility of the network and thus shows good room-temperature self-healing ability. The dynamic mechanical analyzer (DMA) technique was used to study the dynamic mechanical properties of the DCN–PEI polymer. It shows that the DCN–PEI polymer has a high storage modulus of 3370.8 MPa at 25 °C (Figure 4B and Table S1), suggesting excellent rigidity at room temperature. Meanwhile, the transition from the glassy state to the rubbery state takes place well above 25 °C, further confirming the glassy state of the DCN–PEI polymer at room temperature. Moreover, a steady storage modulus stage related to the rubbery state can be observed before 200 °C. It suggests that the DCN–PEI polymer has a covalently cross-linked network, which makes the thermoset DCN–PEI polymer insoluble and infusible at high temperatures. The cross-linking density is calculated to be 354.5 mol/m^3^ according to the equation referring to the rubber-like elasticity theory.^28,29^ It is worth mentioning that this cross-linking density is fairly low in comparison with that of conventional rigid thermoset polymers^30,31^ because the monomer ratio of DCN–PEI that we used is far from the stoichiometric ratio.

Even with low cross-linking density, the DCN–PEI polymer shows a considerably strong mechanical property. Figure 4C shows the tensile stress–strain profiles of the DCN–PEI polymer at an ambient temperature of 21 °C with tensile rates of 1 and 10 mm/min. An abrupt increase in stress of as much as 61.6 MPa and an elastic modulus of as high as 1.6 GPa can be observed and calculated from the initial slope of the stress–strain curve^32^ with a tensile rate of 1 mm/min. In contrast to conventional brittle thermoset polymers, the DCN–PEI specimen yields at an applied strain of 11.2%. The elongation at break is up to 40.3%, and the toughness reaches 19.2 MJ/m^3^. These results demonstrate that the DCN–PEI polymer is mechanically stiff and tough, which is mainly attributed to the weak but abundant and dense hydrogen bonds within the network despite the low covalent cross-linking density. In addition, as shown in Figure 4D, only 1.4 wt % weight loss was detected before 200 °C for the DCN–PEI polymer through the thermogravimetric analysis (TGA) test under air atmosphere, some of which may result from the evaporation of absorbed water molecules. It shows that the DCN–PEI network is thermally stable below 200 °C, demonstrating excellent thermo-oxidative stability.

### 2.2 Room-Temperature Delamination Self-Healing

**Figure 5:**
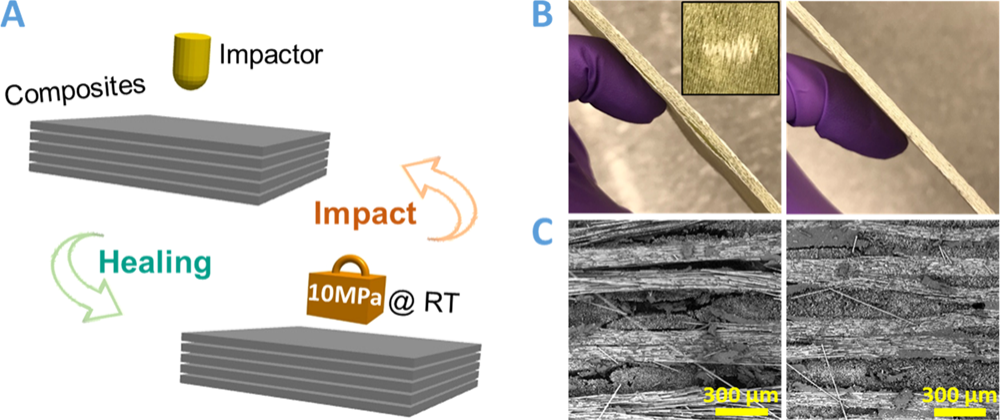
(A) Schematic illustration of the low-velocity impact test and room-temperature self-healing processes. (B) Digital photographs and (C) SEM images of the DCN–PEI based composite laminate before (left) and after (right) room-temperature self-healing for 64 h. The inset image of (B) shows the impact damage zone on the back surface.

As is well known, laminated composites are vulnerable to low-velocity impact load. The high interlaminar shear stress can result in matrix cracking and delamination of the composites, which always reduces the rigidity and strength of the composites.^33,34^ Therefore, here, we perform the low-velocity impact test on glass fiber reinforced DCN–PEI composite laminates to illustrate the room-temperature self-healing properties of pure DCN–PEI. As shown in Figure 5A, the composite specimen was first impacted to create delamination and was then healed upon compression at room temperature for different times. The healed sample was subjected to the low-velocity impact test again at the same location, and the related characteristic parameters were recorded to evaluate the self-healing efficiency. After the impact test, the DCN–PEI composite laminate specimen shows visible delamination and the bending induced matrix cracks toward the back face (Figure 5B), which can be confirmed by the high magnification SEM image (Figure 5C). We can clearly see the wide-opened delamination (∼60 μm) between polymers, indicating the fracture of the DCN–PEI matrix during impact testing. After compression (∼10 MPa) at room temperature for 64 h, the delaminated specimen was healed. Almost no crack can be observed in the high magnification SEM image, suggesting a good healing effect.

**Figure 6:**
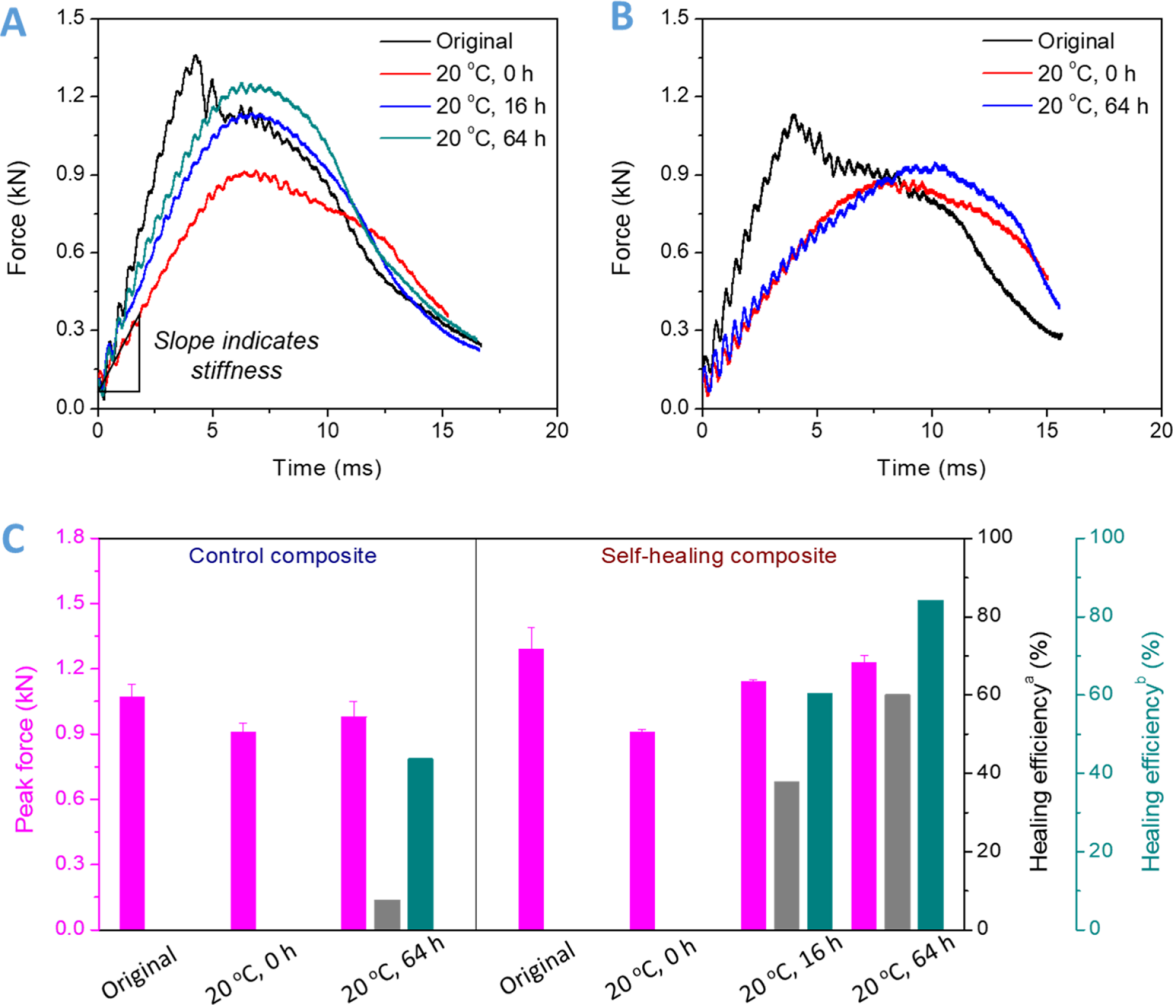
(A) Typical impact load traces of the original DCN–PEI composite laminate and healed DCN–PEI composite laminates at room temperature for different healing times. (B) Impact load profiles of original conventional epoxy resin-based composite laminates and healed conventional epoxy resin-based composite laminates at room temperature for different healing times. (C) Summary of the peak impact force, crack initiation energy, and healing efficiency for control composite laminates (conventional epoxy resin based) and room-temperature self-healable composite laminates (DCN–PEI based). Healing efficiency^a^ was calculated from the force rising rates (initial slope of impact force–time curve), and healing efficiency^b^ was calculated from the recovery of peak impact forces.

The impact forces of the original and healed DCN–PEI based composite laminates were recorded. All the tests follow the same impact parameters, and the same faces and same location were impacted again for these healed specimens. As shown in Figure 6A and Table S2, the impact load of the original specimen reaches 1.29 kN in 4.2 ms, while the unhealed sample (healed for 0 h) only reaches 0.91 kN in a longer time (6.6 ms). It means that the stiffness and strength of the composite laminate are dramatically degraded after the first impact. After healing at 20 °C for 16 and 64 h, the healed specimens gradually build up higher peak forces (1.14 and 1.23 kN, respectively) in a similar period, which indicates the considerable recovery of stiffness and strength. For the purpose of comparison, we used a commercially available strong and tough epoxy thermoset polymer prepared from DGEBA and poly(propylene glycol)bis(2-aminopropyl)ether (Jeffamine D230) to fabricate the control composite laminate, the tensile strength and fracture strain of which reach 70.8 MPa and 15.3% (Figure S6), respectively. The peak load of the control composite laminate is 1.07 kN (Figure 6B), lower than that of the DCN–PEI-based composite laminates, indicating the superiority of the DCN–PEI polymer as the matrix for fabricating high-performance composite laminates. Similarly, the peak load is decreased and time to peak load is increased for the second impact, which suggests the structural failure of the control laminates after the first impact. However, the difference is that the peak load of the control composite was slightly recovered (from 0.91 to 0.98 kN) after the same healing process (20 °C for 64 h), and the force rising rate (slope of the linear region of the impact force–time curve) even remained constant. This suggests that the control thermoset matrix has very little room-temperature self-healing ability.

To qualitatively analyze the self-healing properties, we summarized the impact characteristic values, as shown in Figure 6C and Table S2. First, the self-healing efficiencies were calculated according to peak impact loads before and after healing because they represent the load-bearing ability of composite laminates. The peak load of the original specimen is as the upper boundary, and the peak load of the unhealed specimen is as the lower boundary. The healing efficiencies are calculated based on eq 1. The healing efficiency is calculated to be 43.75 and 84.21% for the control and self-healing composites (healing efficiency^b^ in Figure 6C), respectively. It demonstrates a wide gap of room-temperature self-healing ability between the control and DCN–PEI specimens. The reason that the control laminate also shows healing is that the compression applied during the 64 h of healing at room temperature helps close the delamination, and even some physical entanglement may occur, although no chemical bonding occurs. In addition to using peak impact force as a parameter to evaluate the healing efficiency, other parameters can also be used. For example, due to the initial linear elastic deformation during impact, the initial slope of the impact force–time curve is linear and positively correlated with the slope of the force–deflection curve, and the latter usually represents the stiffness of laminated composites.^35,36^ The stiffness means the resistance to localized elastic deformation of composite laminates and is of great importance in real-world structural applications. Therefore, the healing efficiencies were also determined from the initial slopes of impact force–time curves, as shown in Figure 6C. The control laminate shows a very low healing efficiency of 7.69%, while that of the DCN–PEI composite laminate reaches up to 60.14% (healing efficiency^a^ in Figure 6C). This almost eight times higher healing efficiency suggests the excellent room-temperature self-healing ability of the thermoset DCN–PEI polymer.

### 2.3 Covalent Adaptable Behavior

**Figure 7:**
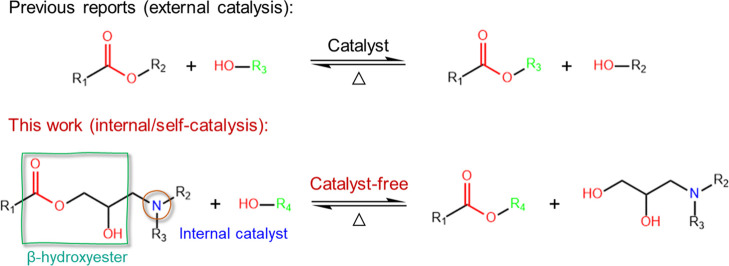
Externally catalyzed transesterification of previous reports and the internal catalysis effect of secondary and tertiary amines of the DCN–PEI network on the transesterification process.

**Figure 8:**
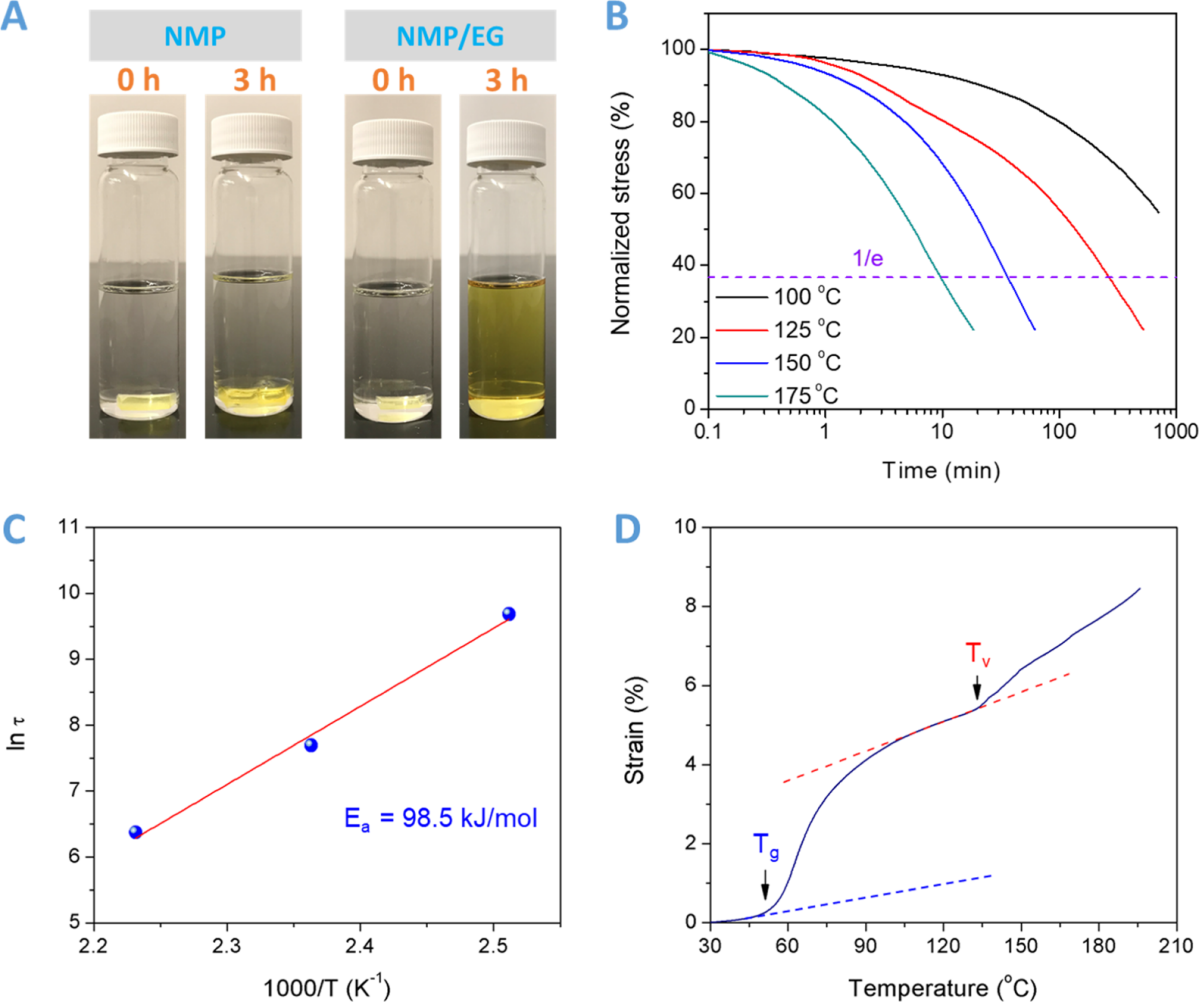
(A) DCN–PEI network is swollen in NMP at 150 °C after 3 h and is dissolved in the NMP/EG mixture at 150 °C after 3 h. (B) Normalized stress-relaxation analysis of the DCN–PEI network. (C) Determination of the relaxation activation energy (*E*_a_) from Arrhenius analysis of the characteristic relaxation time τ vs 1000/*T* for the DCN–PEI network. (D) Temperature dependence of thermal expansion for the DCN–PEI network with a heating rate of 5 °C/min.

For previously reported carboxylic acid/anhydride cross-linked thermoset polymers, external catalysts such as zinc acetylacetonate [Zn(acac)_2_] are always needed due to the fair transesterification rate^1,37^ (Figure 7). In contrast, the well-designed β-hydroxyester and secondary/tertiary amines presented in the DCN–PEI network can accelerate the ester exchange process through the neighboring group effect and internal/self-catalysis action, as reported in previous publications.^38,39^ It makes the use of expensive or toxic catalysts avoidable, which will be an incredible advantage in real-world applications. The dissolution experiments have been conducted to show the solvent resistance of the DCN–PEI polymer. As shown in Figure S7, we can see that the cylinders maintain their shape after 24 h of immersion, indicating that the DCN–PEI polymer did not dissolve in these solvents. They only swell in chloroform, ethylene glycol (EG), DMF, and *N*-methyl-2-pyrrolidone (NMP) after 24 h of immersion and are very stable in acetone, THF, toluene, and hexane. This can be confirmed by the weight changes curves shown in Figure S8. All these results suggest the thermoset characteristic of the DCN–PEI polymer and its good solvents resistance. To study the covalent adaptive network of the DCN–PEI network, two pieces of specimens were immersed into pure NMP and NMP/EG solution, respectively. As shown in Figure 8A, after heating at 150 °C for 3 h, the DCN–PEI specimen is insoluble in pure NMP but sightly swollen, suggesting excellent solvent resistance. By comparison, the DCN–PEI specimen is completely dissolved in the NMP/EG mixture, which demonstrates the rapid transesterification between the DCN–PEI network and EG molecules in the associative mechanism.

Temperature-dependent stress-relaxation tests were also conducted to characterize the dynamic nature of the DCN–PEI network (Figure 8B). In the linear viscoelastic region, 1% strain was applied, and stress decay was recorded as a function of time. The normalized stress of the DCN–PEI network rapidly decreases along with the increase in the relaxing temperature. The characteristic relaxation time (τ) is defined as the time when the normalized stress drops to the value of 1/*e*. It should be noticed that τ at 175 °C is only 9.4 min, indicating fast stress relaxation caused by reconfiguration of the cross-linked network. The linear correlation of ln τ with 1000/*T* demonstrates the Arrhenius flow characteristics of the DCN–PEI network (Figure 8C). The activation energy is calculated to be 98.5 kJ/mol according to the slope of the Arrhenius plot. Creep experiments were performed to monitor the evolution of strain for the DCN–PEI specimen at constant tensile stress (Figure S9). At a high temperature (100 °C), the reversible exchange reaction is activated and the network shows flowability and malleability. After the elastic response, an evident strain increase (∼1.5%) is observed at a tensile stress of 0.1 MPa. While at room temperature, the network is frozen and rigid. Even at higher tensile stress (0.5 MPa), almost no deformation can be detected. This suggests that the topology rearrangements of the adaptable DCN–PEI network are controlled by associative transesterification.

As is well known, the thermal expansion coefficient of permanent cross-linked networks remains constant above *T*_g_, while the topological rearrangement of the covalent adaptive networks always leads to a sudden increase in the expansion coefficient. Thus, the dilatometry test is chosen for distinguishing glass transition and topology freezing transition of covalent adaptive networks.^1,14^ As shown in Figure 8D, the two obvious inflection points can be ascribed to *T*_g_ and topology freezing transition temperature (*T*_v_), respectively. We can see that *T*_g_ is around 50 °C, which is in close agreement with the DSC results. *T*_v_ is determined to be 134 °C. It is much lower than that of the anhydride cross-linked dynamic network (∼195 °C) with 5 mol % external Zn(acac)_2_ catalyst,^1^ confirming the efficient internal catalysis mechanism within the DCN–PEI network. This *T*_v_ value offers guidance on selecting the applicable recycling temperature (150 °C).

### 2.4 Chemical Recyclability of the Carbon Fabric Reinforced DCN–PEI Composite Laminate

**Figure 9:**
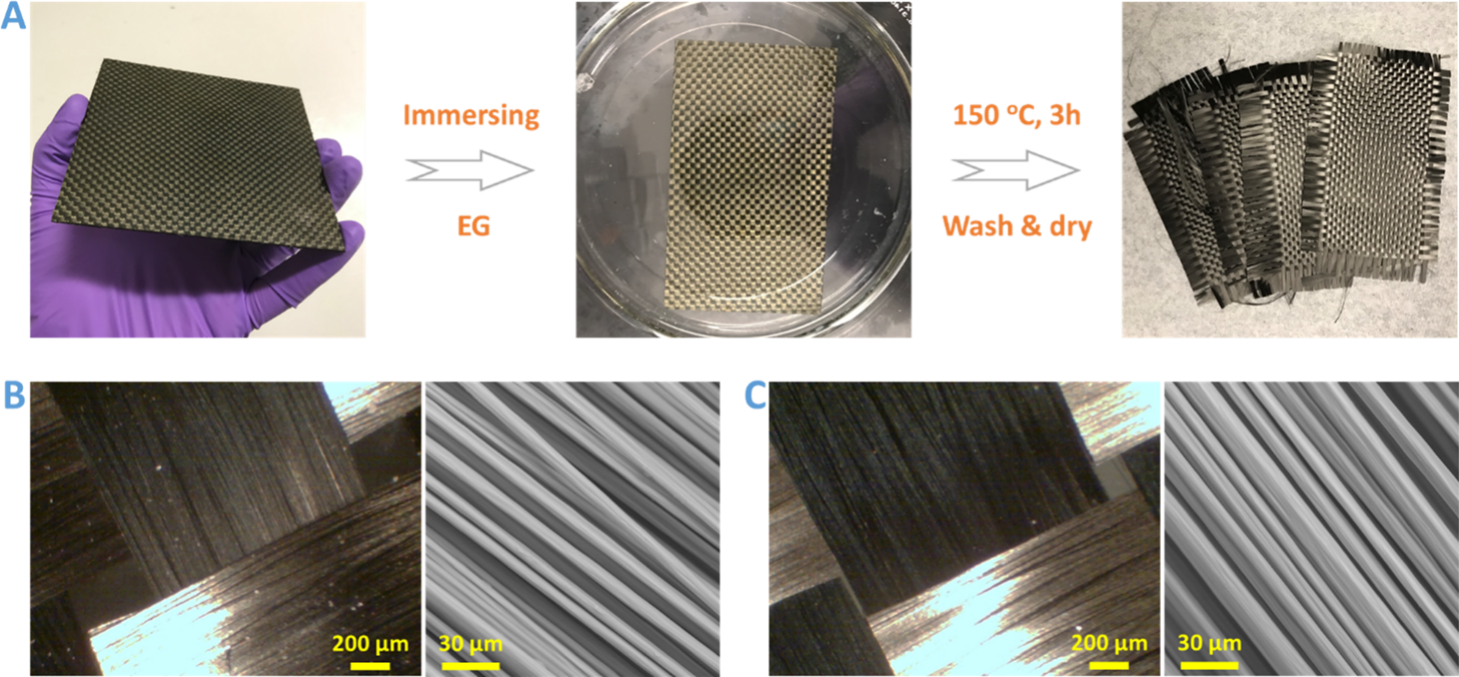
(A) Photographs displaying the chemical recycling process of intact woven carbon fabrics from the DCN–PEI-based composite laminate. Optical microscopy and SEM images of (B) original woven carbon fabrics and (C) chemically recycled woven carbon fabrics.

The strong mechanical properties and dynamic cross-linked network make thermoset DCN–PEI be a promising polymer matrix to fabricate high-performance recyclable carbon fiber reinforced composite laminates. Because of the widespread applications of carbon fibers in reinforcing high-performance load-carrying engineering structures and the high cost of carbon fibers, recycling carbon fiber is of ultimate importance in real-world applications. Figure 9A shows the chemical recycling process of the DCN–PEI-based composite laminate by immersing in EG at 150 °C for 3 h. The polymer matrix was gradually dissolved in EG, and the clean carbon fabric can be separated after washing and drying. The micromorphologies of both the virgin and recycled fabrics were monitored using an optical microscope and SEM, as shown in Figure 9B,C, respectively. The recycled carbon fabric retains the same knitting pattern as the virginal one. No loose fibers can be observed. From high magnification SEM images, we can clearly see that there is no polymer residue attached on the fiber surface and there is no visible damage or alternation in fiber dimension.

**Figure 10:**
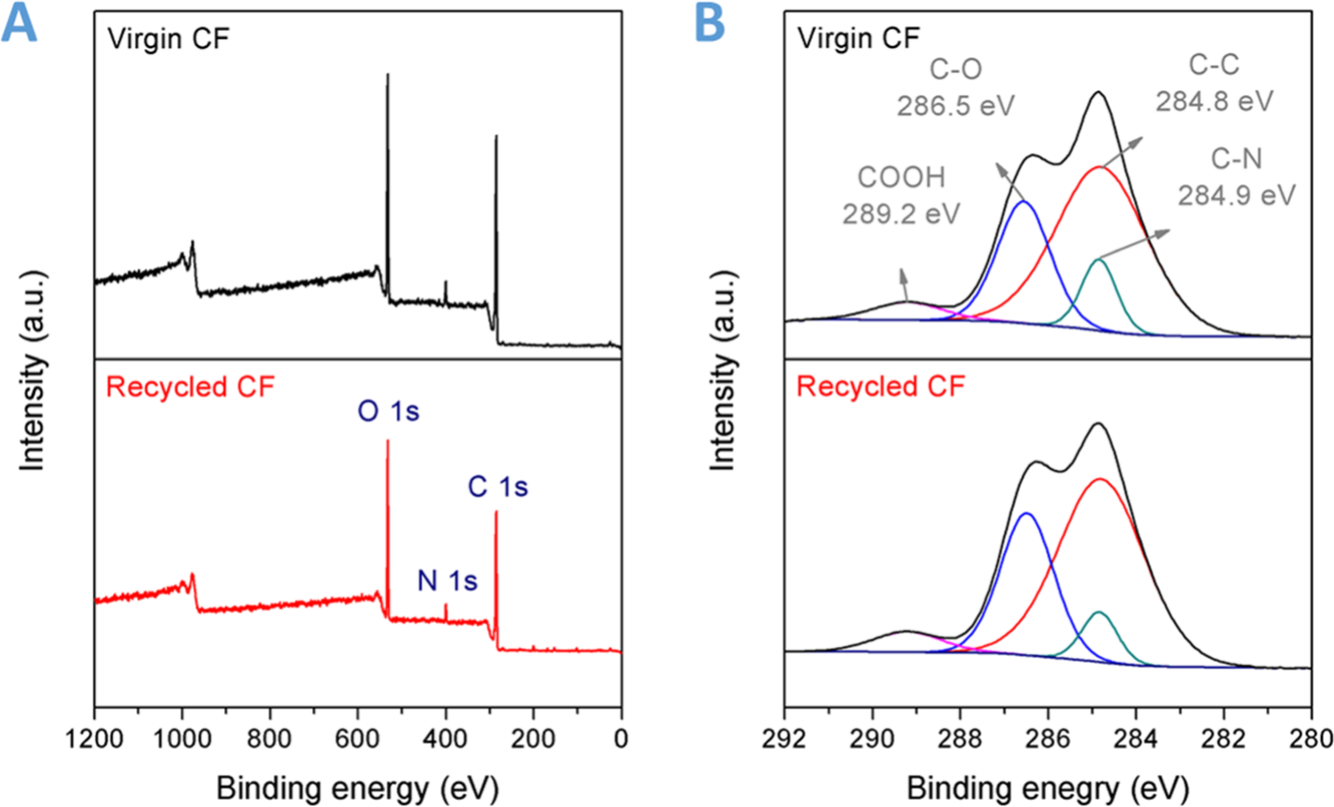
(A) XPS survey spectra and (B) high-resolution C 1s XPS spectra of the virgin carbon fibers and recycled carbon fibers.

Furthermore, the XPS technique was applied to precisely study the surface characteristics of carbon fabrics before and after recycling. Three peaks ascribed to C 1s, N 1s, and O 1s can be observed in the XPS survey spectra of both the virgin and reclaimed fabrics (Figure 10A). It indicates that no impurity element was introduced during the composite fabrication and chemical recycling processes. As shown in Figure 10B, the high-resolution C 1s XPS spectra were deconvoluted into C–C (284.8 eV), C–N (284.9 eV), C–O (286.5 eV), and COOH (289.2 eV). The almost same intensity of each characteristic peak demonstrates that no oxidation or degradation of carbon fabrics occurred, and the DCN–PEI matrix was completely removed during the mild chemical recycling process (Table S3). Moreover, this conclusion can be confirmed by the undifferentiated Raman spectra of the original and reclaimed carbon fabrics (Figure S10).

**Figure 11:**
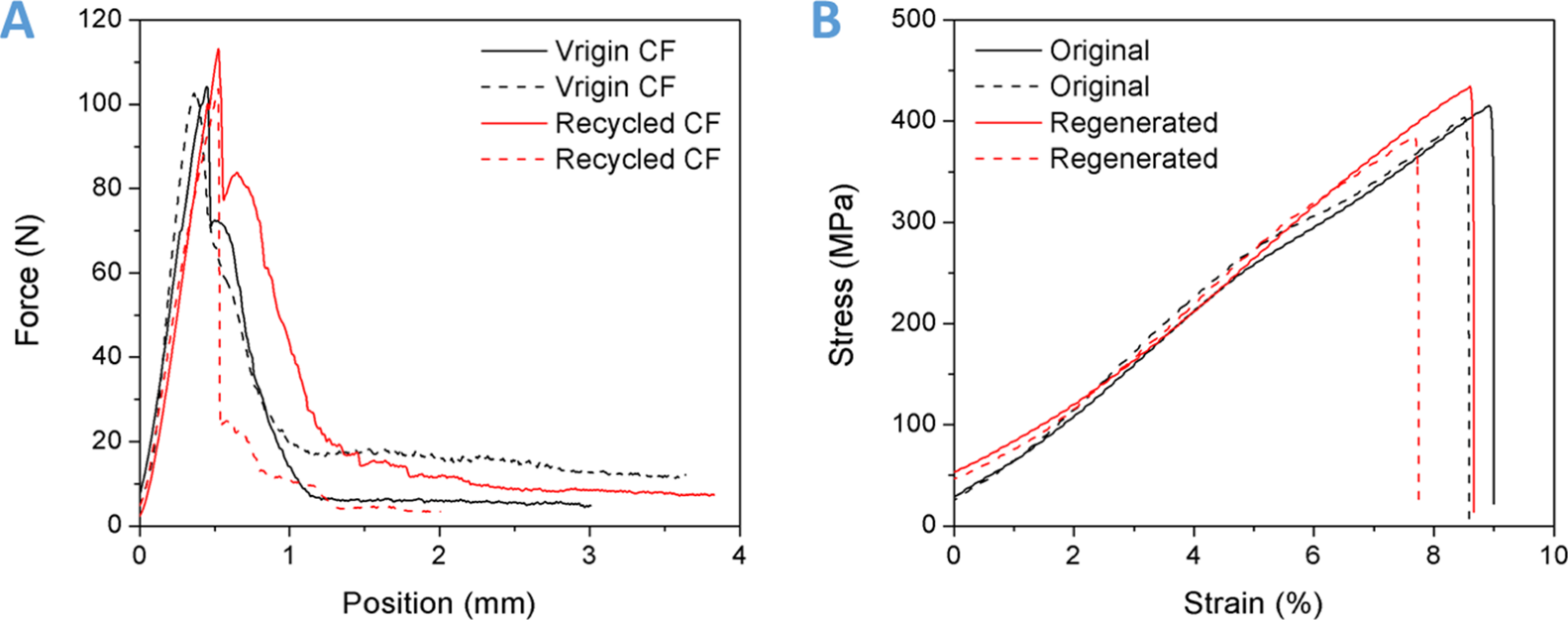
(A) Tensile force and position profiles of the virgin carbon fibers and recycled carbon fibers. (B) Tensile stress–strain curves of the original and regenerated DCN–PEI-based composite laminates.

Tensile tests were performed to evaluate the variation of mechanical properties of the carbon fabrics before and after recycling. Because the carbon fabric is a plain woven one, to eliminate the effect of the fiber bundle that is vertical to the stretching direction, here, we only conduct the uniaxial tension test of a single fiber bundle (∼10 cm in length). Specifically, to be easily clamped by the mechanical testing system (MTS) fixtures, the two ends of the fiber bundles were embedded in an ultraviolet curable acrylate matrix (∼3 mm in thickness and 10 mm in diameter). The stretching rate was set to 1 mm/min in each case. As shown in Figure 11A, the stretching force rapidly increases with the position until a critical value is reached. It demonstrates the fracture of most carbon fibers. The residue force after peak value is primarily caused by the internal friction between each fiber. The good consistency of the force–position (displacement) curves before fracturing suggests that the mechanical properties of the carbon fibers did not degrade during dissolving in noncorrosive EG at a mild recycling temperature (150 °C), which is far below the thermal decomposition temperature (∼500 °C) of carbon fibers; whereafter, the recycled carbon fabrics can be applied to generate DCN–PEI-based composite laminates again. As shown in Figure 11B, the original and regenerated composite specimens show repeatable and comparable tensile stress–strain profiles. The tensile strength (411.0 ± 7.7 MPa) and elongation at break (8.3 ± 0.3%) of the regenerated composite are almost the same as those of the original ones (411.4 ± 7.7 MPa and 8.8 ± 0.3%, respectively) (Table S4), suggesting full recovery of the mechanical performance. These results demonstrate that the DCN–PEI-based composite laminates exhibit great recyclability under mild conditions, and the recycled carbon fibers hold their initial textile structure, surface characteristic, and mechanical properties, which can be reused to fabricate composite laminates without degradation of the reinforcing effect. It is worth mentioning that although we did not show the recycling of the DCN–PEI polymer from the EG solution here, theoretically, the chemically decomposed DCN–PEI matrix can be regenerated by evaporating the EG solvent, as demonstrated in previous reports.^40,41^ Generally, these recycled thermoset polymers show a certain degree of reduction in mechanical strength due to the side reactions during the dissolution and regeneration,^42^ which is not cost-effective, considering the low cost of polymers and the complicated recycling process. Therefore, this study focused on recycling the more expensive carbon fibers.

### 2.5 Performance Comparisons

To achieve room-temperature self-healing ability of glassy polymer-based composites, the most used method is to include external room-temperature curable healing agents into the thermoset polymer matrix stored in some containers, such as microcapsule/microvascular technologies. The resultant room-temperature self-healable polymer composites exhibit the same characteristics as the thermoset matrices, which are stable to solvents but cannot be recycled. Moreover, to achieve acceptable self-healing efficiency, a large number of microcapsules are needed, which always leads to high viscosity of the precursor mixture and degraded mechanical properties of the resultant composites. In addition, the high viscosity and weak microcapsules make it difficult to construct complicated composite structures. Another limitation of using this strategy is that, in general, it can only heal narrow cracks and can only heal the crack one time. Although some new studies have shown promise to heal wider-opened cracks and to heal more than one time, for example, using the high viscosity healing agent and vascular network to deliver the healing agent, the shield life of the healing agent and the manufacturing process present new challenges. Therefore, scientists have been working on developing polymers that can intrinsically heal the cracks at room temperature without adding external healing agents. Most recently, researchers reported very few intrinsic room-temperature self-healable glassy polymers by noncovalently cross-linking low-molecular-weight polymers with dense hydrogen bonds. Unfortunately, the synthesized polymers are thermoplastic. Although thermoplastics are intrinsically recyclable, they are unstable to thermal and chemical solvents. Furthermore, their mechanical properties are generally unsatisfactory for load-bearing structures because of the weak noncovalent cross-linking. In contrast, our room-temperature self-healable DCN–PEI network is covalently cross-linked, which makes it infusible and insoluble. In addition, the self-catalyzed associative transesterification mechanism endows it with desirable recyclability under mild conditions. It means that the DCN–PEI polymer is the first glassy thermoset polymer that integrates intrinsic room-temperature self-healing ability and recyclability.

**Figure 12:**
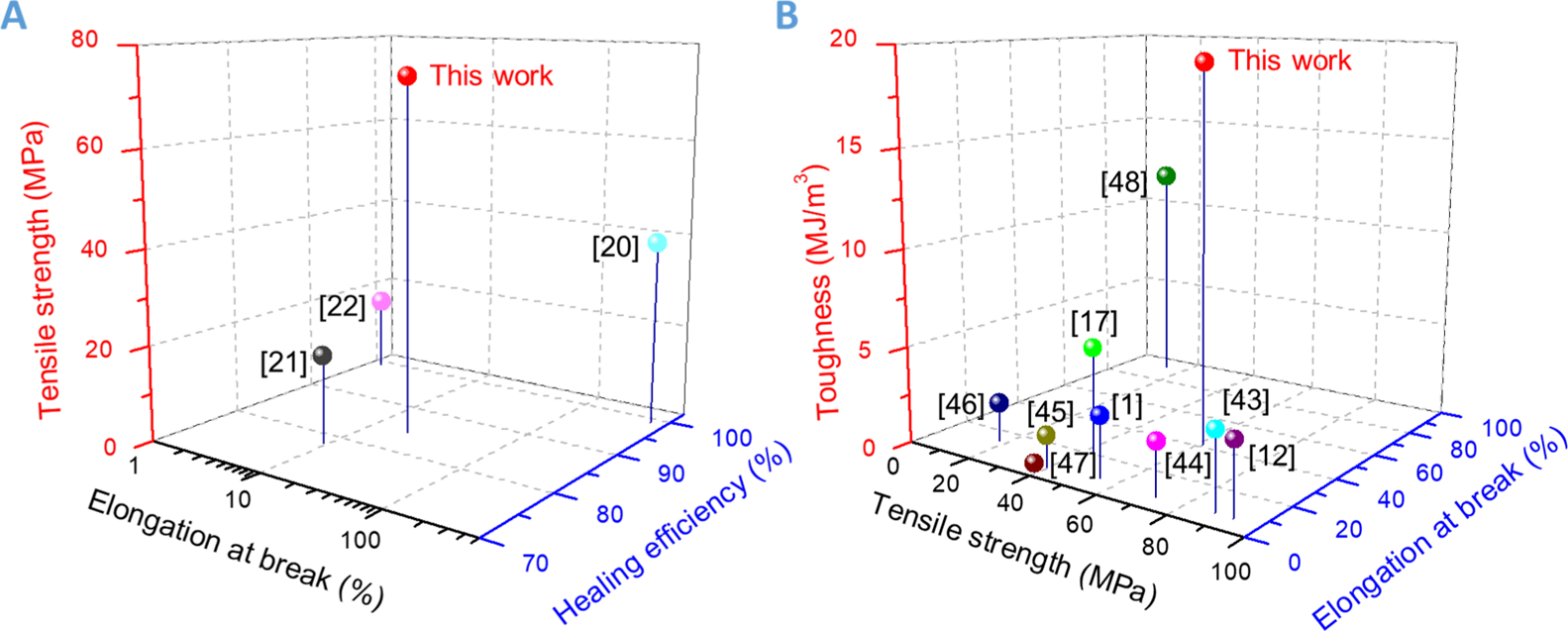
(A) Comparison to the few reported intrinsic room-temperature self-healing glassy polymers. The thermoset DCN–PEI polymer exhibits outstanding tensile strength and acceptable healing efficiency. (B) Toughness vs tensile strength vs elongation at break chart showing that the DCN–PEI polymer has exceptional toughness over the published covalent adaptable networks.

Most importantly, as shown in Figure 12A, due to the dual cross-linking network (covalent and noncovalent), our DCN–PEI polymer exhibits outstanding mechanical strength (73.9 MPa, tested at a stretching rate of 10 mm/min), which is the highest among the reported room-temperature self-healable glassy polymers (data tested at the same stretching rate of 10 mm/min).^20−22^ Furthermore, the mechanical properties of the DCN–PEI network were compared with those of previously reported recyclable thermoset polymers^1,12,17,43−48^ (Figure 12B). We can see that the tensile strength of the DCN–PEI polymer is above average, and the elongation at break is higher than that of most references. The combination of strong tensile strength and high elongation at break endows the recyclable DCN–PEI polymer with state-of-the-art toughness. These promising strong mechanical properties, room-temperature self-healing ability, and chemical reprocessability are attributed to the optimal and balanced dual-cross-linked network of DCN–PEI. The proper dynamic covalent cross-linking density endows the vitrimer with thermoset features, high mechanical strength, and exceptional reprocessability. The noncovalent cross-linking through dense hydrogen bonds endows the vitrimers with excellent toughness, and the high mobility of the side chains in the branched network structures facilitates the room-temperature self-healing performance. The construction of this unique network relies on the branched structure of PEI cross-linkers and the inherent carboxylate ester of DCN monomers, and most importantly, the balance between the dynamic covalent cross-linking and noncovalent cross-linking. Although lowering the glass transition temperature can enhance the hydrogen bonds and thus the room temperature healing efficiency, it reduces the room temperature mechanical strength and stiffness and vice versa.

## 3 Conclusions

In summary, here, we propose a dual cross-linking strategy of low-molecular-weight branched PEI with an ester-containing epoxy monomer to construct a room-temperature self-healing glassy thermoset polymer network. The network combines weak but dense noncovalent hydrogen bond cross-linking and strong but sparse covalent cross-linking. These two complementary cross-linking actions contribute to a high tensile strength of 61.6 MPa, an elastic modulus of 1.6 GPa, and a toughness of 19.2 MJ/m^3^ for the resulted polymer, as well as good thermal and chemical stabilities. Due to the branched molecular structures, the multiple hydrogen bonds formed between the branching units and terminal groups exhibit high mobility and exchange capability, which imparts room-temperature self-healing ability to the polymer although the major network is frozen. The self-healing efficiency was determined to be 84.21% through the low-velocity impact test of the polymer-based composite laminates. Furthermore, the presence of β-hydroxyester and the internal catalytic action of the secondary/tertiary amines enable the thermoset polymer to be readily dissolved in alcohols through a catalyst-free transesterification process at mild temperature. The recycled carbon fibers from composite laminates possess the same structures and strengths as the initial one, which can reinforce the laminates again without degradation of mechanical performance.

## 4 Experimental Section

### 4.1 Synthesis of the DCN–PEI Thermoset Polymer

Figure 1A shows the mechanism used to create the DCN–PEI thermoset polymer (a mass ratio of 1/1). At room temperature, the branched PEI (average *M*_w_ ∼ 800 by LS, average *M*_n_ ∼ 600 by GPC, Sigma-Aldrich) and DCN (Sigma-Aldrich) were mixed equally in quantity in an aluminum weighing dish, and then, the homogenous mixture was degassed in a vacuum oven for 30 min to completely remove the bubbles. The obtained clear mixture was allowed to cure at 100 °C for 2 h and post-cure at 150 °C for another 2 h. The resulted thermoset polymer sample was kept in a vacuum desiccator before the various tests and is abbreviated as DCN–PEI in the main text or DCN–PEI-1/1 in Supporting Information. Moreover, the networks with different ratios (2/1, 1.1/1.2, and 1/1.5) of DCN to PEI were fabricated following the same procedure except for the different reactant ratios. The obtained samples are abbreviated as DCN–PEI-2/1, DCN–PEI-1.1/1.2, and DCN–PEI-1/1.5 in Supporting Information.

### 4.2 Fabrication of Composite Laminates

50 g of branched PEI and 50 g of DCN were mixed and degassed into a homogeneous mixture at room temperature. For studying the recyclability, woven carbon fabric was used to reinforce the DCN–PEI matrix. The DCN–PEI-based composite laminates were fabricated by hand applying the clear resin solution onto four layers of woven carbon fabric from Fibre Glast and then hot-pressing them into 1 mm of thickness between two aluminum foils. The curing process was achieved at 100 °C for 2 h and at 150 °C for another 2 h. The final specimens were obtained by peeling off the aluminum foils and cutting away the edges. The volume fraction of the carbon fabric in the composite laminate is calculated to be 51.5%. The regenerated carbon fabric reinforced DCN–PEI-based composite laminates were fabricated by the same method except for the usage of recycled woven carbon fabric.

For studying the room-temperature self-healing of delamination, woven glass fiber reinforced polymer composite laminates were fabricated. Two types of polymers were used, one is our DCN–PEI polymer, and the other is a conventional epoxy thermoset that was prepared by cross-linking DGEBA (epoxide equivalent weight, 172–176, Sigma-Aldrich) with poly(propylene glycol) bis(2-aminopropyl ether) (average *M*_n_ ∼ 230, Sigma-Aldrich) in a stoichiometric ratio as the control. For preparing the woven glass fabric reinforced composite laminates, eight layers of woven glass fabric were used, and the thickness of the laminates was set to be 2.5 mm. The volume fraction of the glass fabric in the composite laminate is calculated to be 42.4%. All the fabrication parameters are the same except for the usage of different thermoset polymer matrices. The curing condition for control composite laminates is at 100 °C for 2 h and at 130 °C for another 2 h.

### 4.3 Characterization

The FTIR spectra were recorded using a Nicolet 6700 FTIR spectrometer (Thermo Fisher Scientific, USA) in attenuated total reflection mode by collecting 32 scans from 500 to 4000 cm^–1^. The curing kinetics and glass transition temperature (*T*_g_) were studied by a PerkinElmer 4000 differential scanning calorimeter DSC (MA, USA). The purging rate of nitrogen gas was 30 mL min^–1^. For the curing kinetics test, 5–10 mg of the monomer mixture was heated from −50 to 220 °C at different heating rates (2.5, 5, 10, and 20 °C/min). For determining *T*_g_, the samples were heated and cooled between −50 and 200 °C at three different heating/cooling rates of 5, 10, and 15 °C/min; both the holding times at −50 and 200 °C were 2 min. The second heating–cooling cycle was conducted and used to determine *T*_g_. The TGA profiles were obtained by using a Q5000 thermal analyzer (TA Co., USA) from 20 to 700 °C at a heating rate of 10 °C/min in both argon and air atmospheres. The purging rate of gas was 100 mL min^–1^. The tensile test was conducted using an eXpert 2610 MTS (ADMET, Norwood, MA, USA). The stretching rate was 1 mm/min for the composite laminate specimens and carbon fibers before and after recycling. Both 1 and 10 mm/min were performed to characterize the tensile properties of the neat DCN–PEI. At least three parallel samples were performed for tensile tests. The micromorphologies of the composite laminates and woven carbon fabrics were characterized by an optical microscope (AmScope MD35) and a scanning electron microscope (JSM-6610 LV, JEOL, USA) at an operation voltage of 5 kV, respectively. The XPS spectra were recorded using a Scienta Omicron ESCA 2SR X-ray photoelectron spectroscope. A Renishaw inVia reflex Raman spectroscope was utilized to study the structure of carbon fabrics before and after recycling. The excitation wavelength was 532 nm, and the Raman shift was scanned from 200 to 4000 cm^–1^. The dynamic mechanical properties of the PEI–DCN network were evaluated using a Q800 DMA (TA Instruments, DE, USA) in multifrequency strain mode at a heating rate of 3 °C/min and a frequency of 1 Hz. For the stress relaxation test, the rectangular specimens were first equilibrated at 100, 125, 150, and 175 °C for 30 min and were then stretched to a constant strain of 1.0%. The stress value was recorded over time. For creep testing, after thermally equilibrating at room temperature or 100 °C for 30 min, the rectangular specimens were stretched by a constant stress and held for 20 min. The strain increase was recorded over time. The dilatometry experiment was conducted by the DMA apparatus in tension film geometry. The length was monitored while heating from 30 to 200 °C at a rate of 5 °C/min.

The low-velocity impact tests were conducted using an Instron Dynatup 8250 H V impact tester according to ASTM standard D3763-18. The composite specimens (14.5 cm × 4.5 cm × 0.25 cm) were impacted by a hammer weight of 11.2 kg that was dropped from a height of 20 cm. After impact tests, the damaged samples were collected and stored in the sealed plastic ziplock bags, and the self-healing experiments were conducted within 12 h. The room-temperature self-healing of the composite laminates was achieved by compressing the impacted specimens under ∼10 MPa at room temperature for different times (0, 16, and 64 h). The healed specimens were subjected to the low-velocity impact test again at the same location on the same surface. The self-healing efficiency was calculated according to the following equation
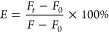
1where *E* is the healing efficiency, *F* is the peak impact force of the original specimen, *F*_0_ is the peak impact force of the specimen with zero-hour self-healing time, and *F*_*t*_ is the peak impact force of the specimen with *t* hours of self-healing time, here 16 h or 64 h. For calculating healing efficiency according to force rising rates, *F*, *F*_0_, and *F*_*t*_ are initial slopes of the force–time curves for the original specimen, the specimen with zero-hour self-healing time, and the specimen with *t* hours of self-healing time (16 h or 64 h), respectively.

Chemical recycling of carbon fibers from the carbon fabric reinforced DCN–PEI composite laminates was conducted by immersing the intact composite plate in 200 mL of EG at 150 °C for 3 h. After completely dissolving the DCN–PEI matrix, the dissociated woven carbon fabrics were then collected, gently washed with ethyl alcohol five times, and naturally dried in a hood. The recycled carbon fabrics were then used to fabricate the regenerated DCN–PEI composite laminates.

## Supporting Information Available

DSC heating curves and curing kinetics of DCN/PEI mixtures, FTIR spectra of the cross-linked DCN–PEI network, glass transition temperatures of DCN–PEI networks determined by DSC, TG, and DTG profiles of DCN–PEI under nitrogen atmosphere, room-temperature tensile stress–strain profile of the control epoxy thermoset polymer, creep behaviors of DCN–PEI at room temperature and high temperature, basic properties of the DCN–PEI network determined through DMA, summary of room-temperature self-healing properties and chemical recyclability of DCN–PEI-based composite laminates, XPS results of original and recycled carbon fibers, and tensile properties of the original and regenerated DCN–PEI-based composite laminates (PDF)

## Notes

The data that support the findings of this study are available from the corresponding authors upon reasonable request.
