# Supplementary material for: Room-Temperature Self-Healable and Mechanically Robust Thermoset Polymers for Healing Delamination and Recycling Carbon Fibers
Source: ACS Appl Mater Interfaces. 2021 Oct 27;13(44):53099–110. doi: 10.1021/acsami.1c16105 (PMC8587616; doi:10.1021/acsami.1c16105)
Supplement: Supplementary file 1 — am1c16105_si_001.pdf [file am1c16105_si_001.pdf]

Supporting Information

Room-Temperature Self-Healable and Mechanically Robust Thermoset Polymer for Healing Delamination and Recycling Carbon Fiber

Xiaming Feng, Guoqiang Li*

Department of Mechanical & Industrial Engineering, Louisiana State University, Baton Rouge, LA 70803, United States

**Corresponding author. E-mail: lguoqi1@lsu.edu; Tel.: 001-225-578-5302*

Calculation of activation energy (E_a) from non-isothermal curing scans

According to Kissinger's method,[1] the curing activation energy can be calculated from the peak temperature at different heating rates following Eq. (S1):

$$ln\left(\frac{\beta}{T_p^2}\right) = -\frac{E_a}{RT_p} + ln\left(\frac{AR}{E_a}\right) \qquad \text{(S1)}$$

where T_p represents exothermic peak temperature, β is heating rate of the non-isothermal curing scan, A demotes a pre-exponential factor and R is universal gas constant (8.314 $m^3 \cdot Pa/K \cdot mol$). The activation energy E_a is calculated to be 63.5 kJ/mol.

Determination of cross-linking density (δ) of DCN-PEI network

Cross-linking density can be figured out using Eq. (2):[2-3]

$$\delta = E'/3RT \qquad \text{(S2)}$$

where R is universal gas constant (8.314 $m^3 \cdot Pa/K \cdot mol$); T is absolute temperature (395.85 K), and E' is storage modulus (3.5 MPa) at rubbery state (T_g + 50 °C). The δ value for PEI-DCN network is calculated to be 354.5 mol/m^3.

Calculation of activation energy (E_a) for DCN-PEI covalent adaptive network

The relaxation time τ can be fitted to the Arrhenius equation (Eq. (3)):[4-5]

$$\ln \tau\,(t) = \ln \tau_0 + E_a\,/RT \qquad \text{(S3)}$$

where R is universal gas constant (8.314 $m^3 \cdot Pa/K \cdot mol$); T is absolute temperature; the characteristic relaxation time τ is determined from the stress relaxation tests at different temperatures (Figure 4C). τ_0 is a constant. Three data pairs (lnτ, 1/T) were fitted by linear regression as follow:

$$y = 11.85\ x - 20.16 \qquad \text{(S4)}$$

where x is 1000/T (K^{-1}) and y is ln τ (s). From Eqs. (S3) and (S4), the activation energy E_a is calculated to be 98.5 kJ/mol.

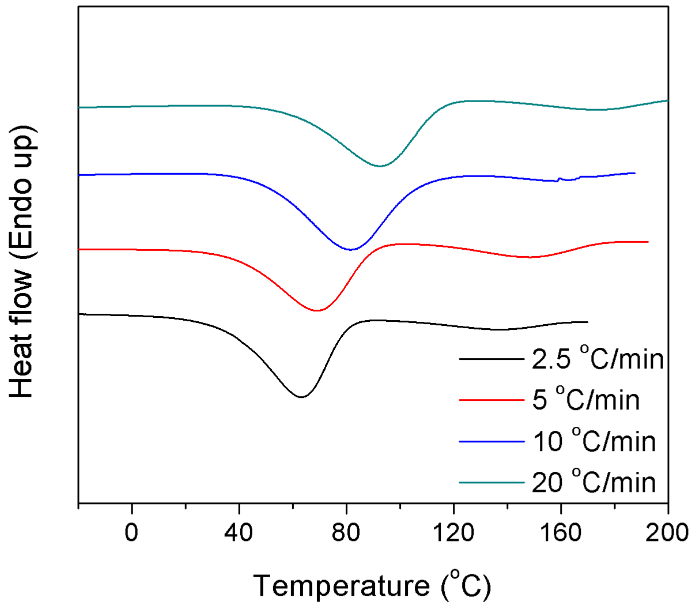

Figure S1. DSC heating curves of DCN/PEI mixture at different heating rates.

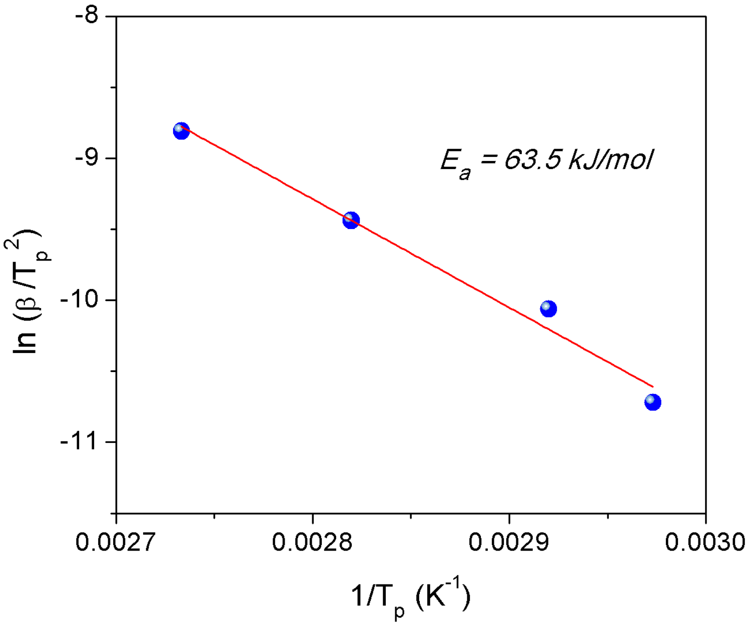

Figure S2. A linear plot of ln (β/T_p^2) versus $1/T_p$ according to Kissinger's method.

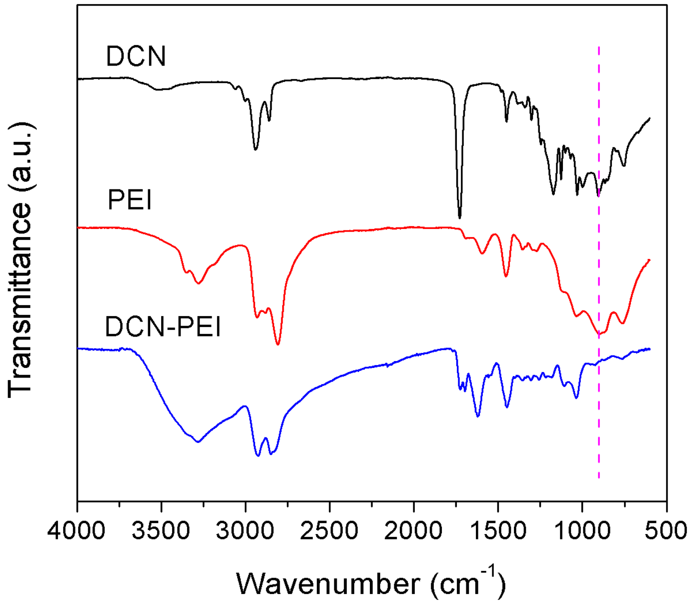

Figure S3. FTIR spectra of the DCN, PEI monomers and crosslinked DCN-PEI polymer.

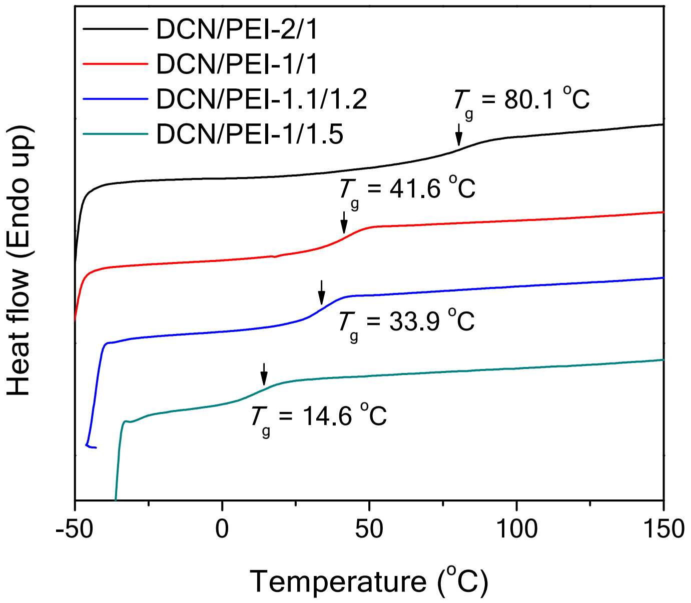

Figure S4. DSC profiles for DCN-PEI polymer on the second heating from –50°C to 150°C at different weight ratios of DCN/PEI monomers.

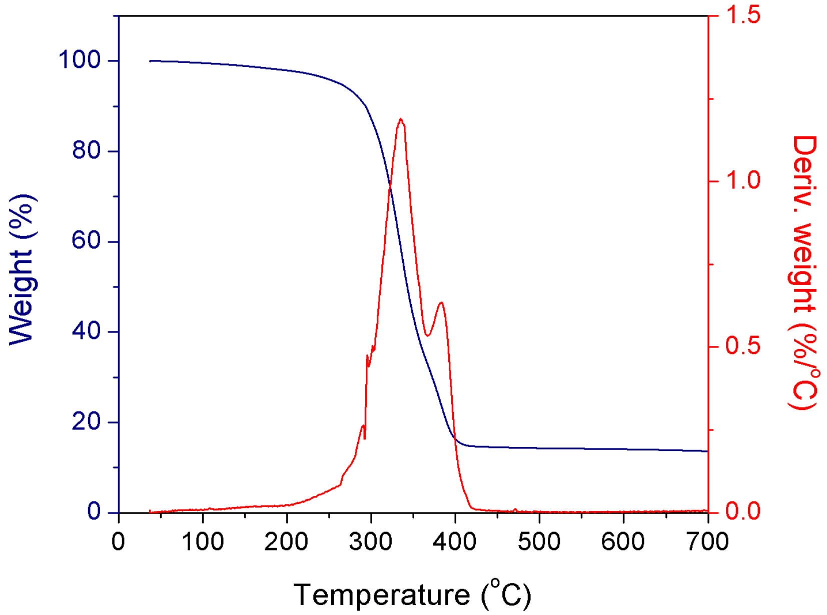

Figure S5. TG and DTG curves of DCN-PEI in N_2 atmosphere at a heating rate of 10 °C/min.

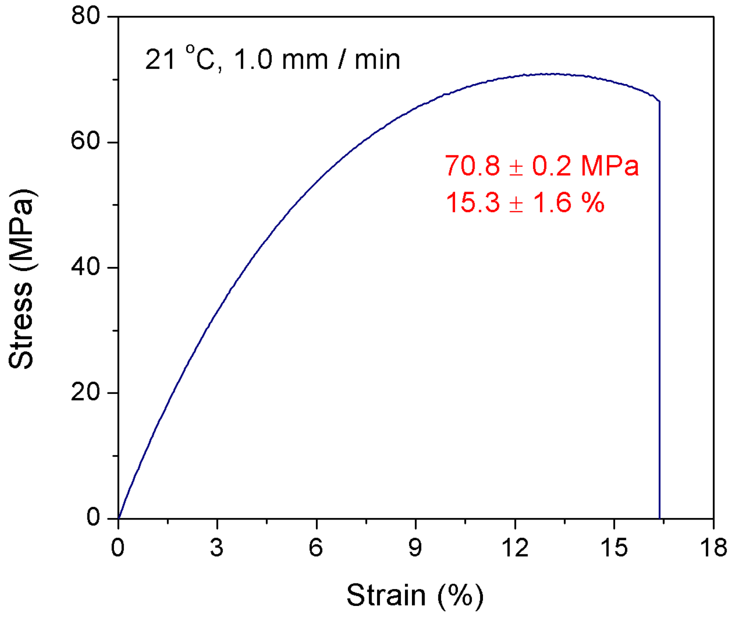

Figure S6. Room-temperature tensile stress-strain profile of the control epoxy thermoset polymer prepared from DGEBA and Jeffamine D230 at a stretching rate of 1 mm/min.

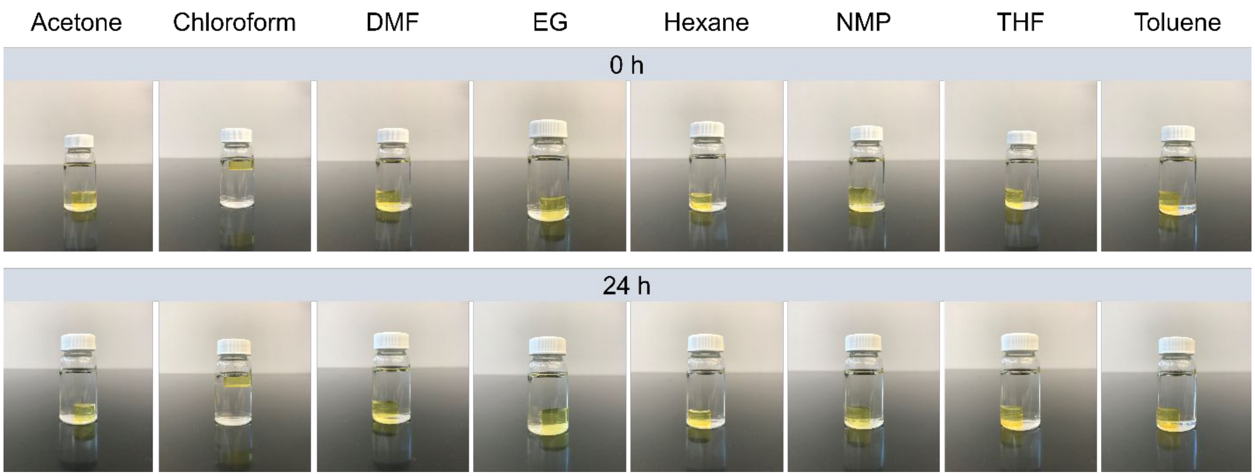

Figure S7. Dissolution experiments for the DCN-PEI samples in various solvents at room temperature for 24 h.

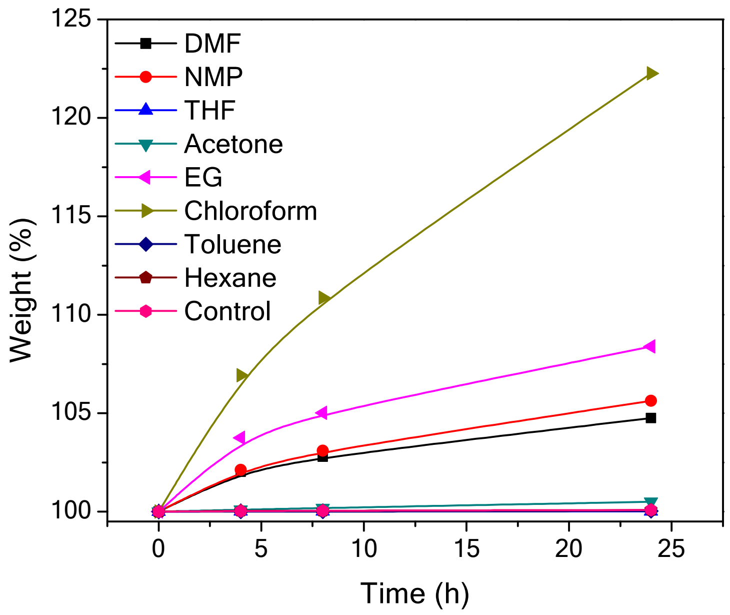

Figure S8. Weight changes of the DCN-PEI specimens in various chemical solvents at room temperature.

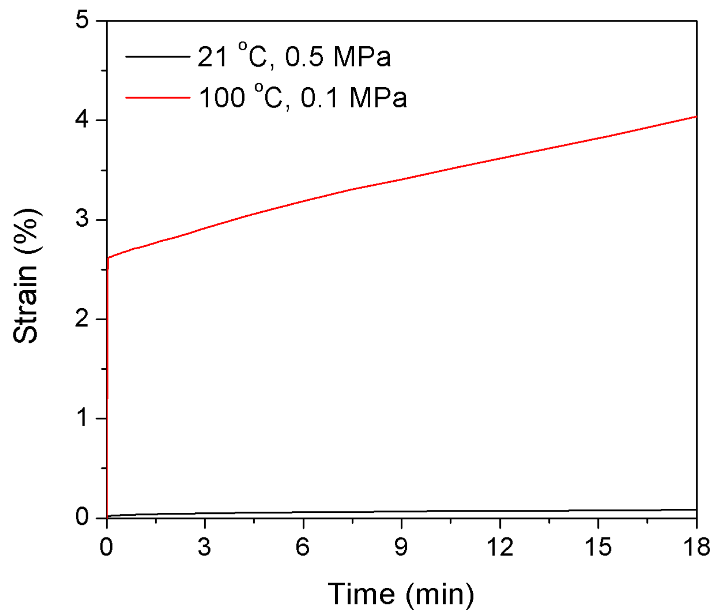

Figure S9. Creep performance of DCN-PEI sample at different temperatures and different stretching loads.

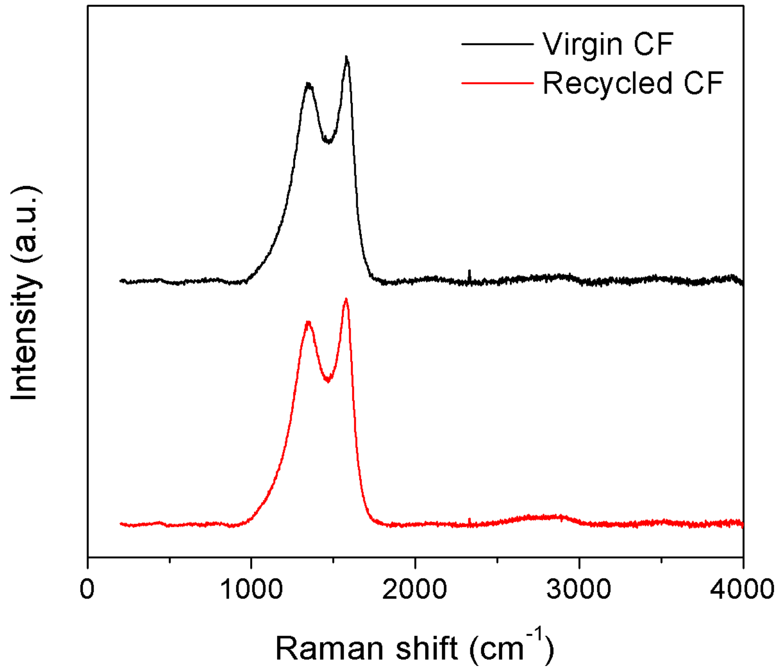

Figure S10. Raman spectra of the virgin carbon fibers and recycled carbon fibers.

Table S1. Basic properties of DCN-PEI network determined through DMA.

**Sample**	**T_g (°C)**	**Glassy modulus (25 °C) (MPa)**	**Rubbery modulus (T_g + 50 °C) (MPa)**	**Crosslinking density (mol/m^3)**
DCN-PEI	72.7	3370.8	3.5	354.5

Table S2. Room-temperature self-healing properties of DCN-PEI based composite laminates and control composite laminates evaluated by low-velocity impact test.

Sample	Peak force (kN)	Healing efficiency[a] (%)	Healing efficiency[b] (%)
DCN-PEI	1.29 ± 0.10	--	--
20 °C, 0 h	0.91 ± 0.01	--	--
20 °C, 16 h	1.14 ± 0.01	37.84	60.53
20 °C, 64 h	1.23 ± 0.03	60.14	84.21
Control	1.07 ± 0.06	--	--
20 °C, 0 h	0.91 ± 0.04	--	--
20 °C, 64 h	0.98 ± 0.07	7.69	43.75

[a] Healing efficiency calculated from the force rising rates (initial slope of impact force-time curve).

[b] Healing efficiency calculated from the recovery of peak impact forces.

Table S3. The atomic percentage of each element in virgin and recycled carbon fabrics tested by XPS.

Sample	C 1s	N 1s	O 1s
Virgin	72.1 at. %	3.9 at. %	22.7 at. %
Recycled	70.4 at. %	3.8 at. %	23.0 at. %

Table S4. Tensile properties of the original and regenerated DCN-PEI based composite laminates.

Sample	Tensile strength (MPa)	Elongation at break (%)
Original	411.4 ± 7.7	8.8 ± 0.3
Regenerated	411.0 ± 34.7	8.3 ± 0.6

References

(1) Kissinger, H. E. Variation of Peak Temperature with Heating Rate in Differential Thermal Analysis. *J. Res. Natl. Bur. Stand.* **1956,** *57*, 217-221.

(2) Moore, C.; Scanlan, J. Determination of Degree of Crosslinking in Natural Rubber Vulcanizates. Part VI. Evidence for Chain Scission during the Crosslinking of Natural Rubber with Organic Peroxides. *J. Polym. Sci.* **1960,** *43*, 23-33.

(3) Hill, L. W. Calculation of Crosslink Density in Short Chain Networks. *Prog. Org. Coat.* **1997,** *31*, 235-243.

(4) Montarnal, D.; Capelot, M.; Tournilhac, F.; Leibler, L. Silica-Like Malleable Materials from Permanent Organic Networks. *Science* **2011,** *334*, 965-968.

(5) He, C. F.; Shi, S. W.; Wang, D.; Helms, B. A.; Russell, T. P. Poly(oxime-ester) Vitrimers with Catalyst-Free Bond Exchange. *J. Am. Chem. Soc.* **2019,** *141*, 13753-13757.
